# Association Between Life Expectancy at Age 60 Years Before the COVID-19 Pandemic and Excess Mortality During the Pandemic in Aging Countries

**DOI:** 10.1001/jamanetworkopen.2022.37528

**Published:** 2022-10-19

**Authors:** Mitsuyoshi Urashima, Emiri Tanaka, Hiroto Ishihara, Taisuke Akutsu

**Affiliations:** 1 Division of Molecular Epidemiology, Jikei University School of Medicine, Tokyo, Japan

## Abstract

This cross-sectional study investigates the association between life expectancy before COVID-19 and excess mortality during the pandemic in aging countries.

## Introduction

The World Health Organization (WHO) estimated that the COVID-19 pandemic has been associated with approximately 15 million excess deaths.^1^ Excess mortality (EM) from all causes per 100 000 population members in each country varied from negative values to more than 300 deaths. Older age is one of the factors associated with the greatest increase in risk of COVID-19 deaths,^2^ suggesting that EM is likely to be high in aging countries. However, although Japan has the highest aging ratio in the world, it was able to keep EM low during the pandemic. To investigate this contradiction, we explored associations of health, well-being, population, and economic factors before the pandemic with EM during the pandemic.

## Methods

This cross-sectional study followed the STROBE reporting guideline. The Jikei University School of Medicine determined that institutional review board approval and informed consent were not required due to the use of publicly available data from open sources without personal information.

An ecological study design was applied to analyze the strength of associations between 51 covariates in individual countries before the COVID-19 pandemic (eTable in the Supplement) and EM during the pandemic from January 2020 to December 2021, as presented by WHO.^1^ Covariates included life expectancy at age 60 years as measured in 2016. Simple linear regression models were used to screen factors associated with EM. A 2-sided *P* < .001 was considered significant. The Pearson correlation coefficient for variables (ρ) was used to quantify strengths of correlations. Data were analyzed using Stata statistical software version 17.0 (StataCorp).

## Results

**Figure 1.**
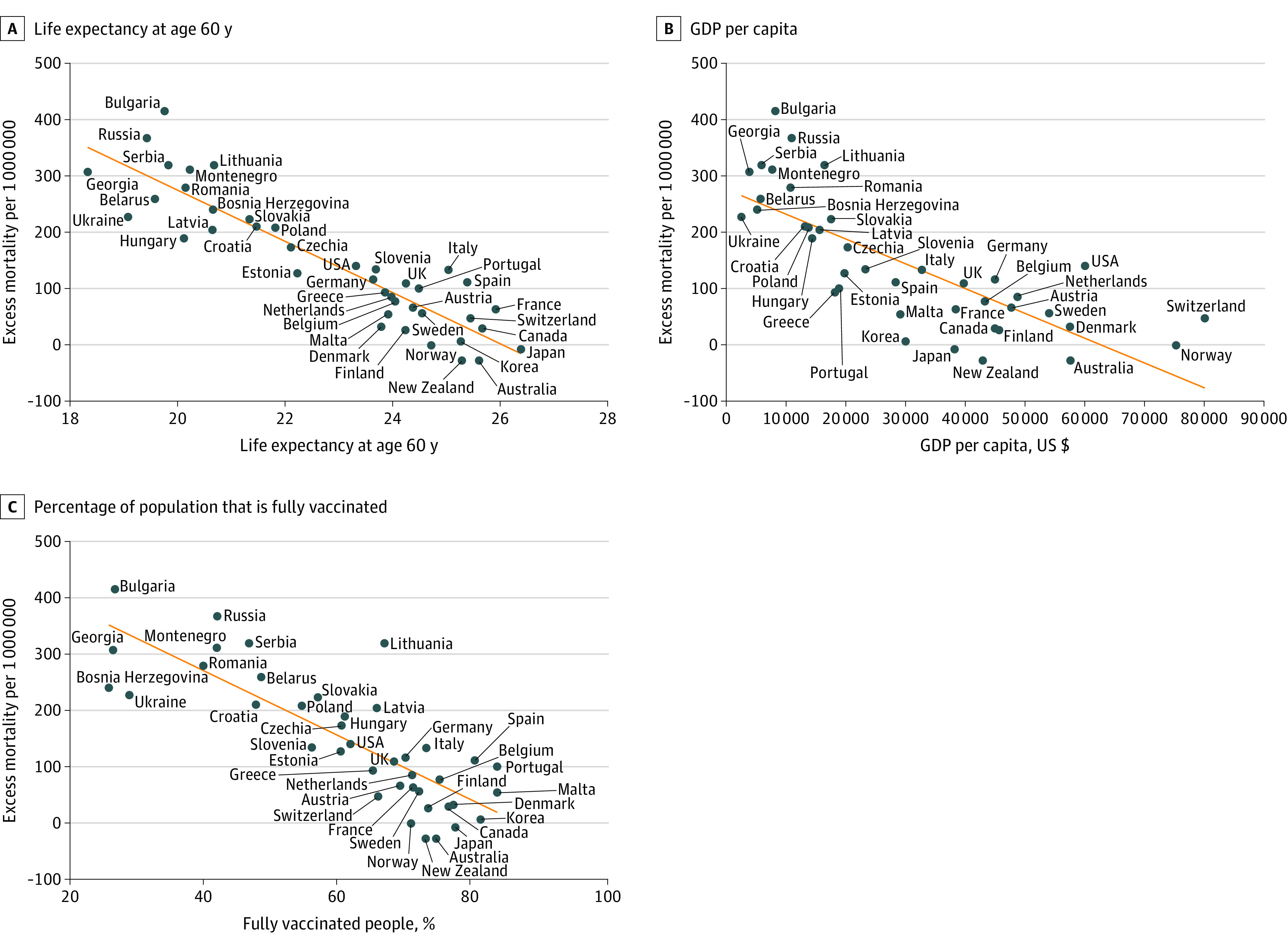
Association Between Population-Derived Factors and Excess Mortality During COVID-19 Pandemic Associations are presented in 40 aging countries (quartile 4).

**Figure 2.**
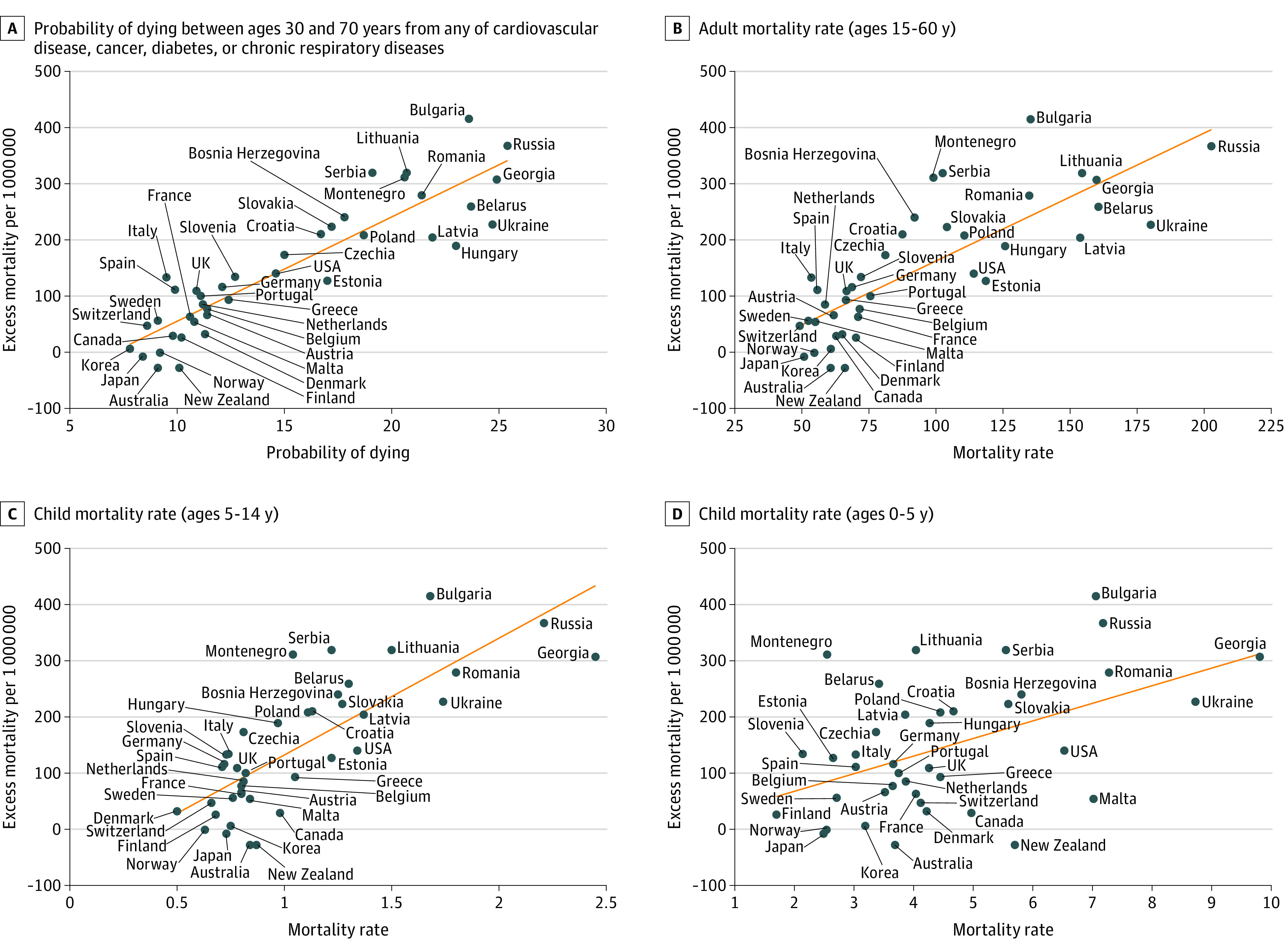
Association Between Mortality by Age Group and Excess Mortality During COVID-19 Pandemic

The median proportion of the population aged 60 years or older in 158 countries analyzed was 9.7%, ranging from 2.8% to 34.0%, and was divided into quartiles. In 40 aging countries (quartile 4), but not in others (quartiles 1-3), after excluding factors that were significant due to collinearity, 3 factors had associations with EM and ρ values that were strong or higher (ie, ≥0.7): life expectancy at age 60 years (ρ = −0.91), gross domestic product (GDP) per capita (ρ = −0.78), and the percentage of fully vaccinated people in the population (ρ = −0.82) (Figure 1), all of which remained after adjustment for mean age of the country. In multiple linear regression analysis, only life expectancy at age 60 years remained statistically significant. Among mortality rates by age group, probability of dying from any of cardiovascular disease, cancer, diabetes, or chronic respiratory disease at ages 30 to 70 years had the strongest association with EM (ρ = 0.90). However, mortality rates in adults (ages 15-60 years; ρ = 0.80) and children (ages 5-14 years; ρ = 0.78) had weaker associations with EM, and the mortality rate for individuals aged 0 to 5 years had no association with EM (Figure 2).

## Discussion

In this cross-sectional study, an association was observed between life expectancy at age 60 years before the COVID-19 pandemic and EM during the pandemic only in aging countries (quartile 4) even after adjustment for GDP and vaccination. Moreover, prepandemic mortality in adults aged 30 to 70 years was associated with EM in quartile 4, but mortality among individuals aged 5 years or younger was not. These findings are similar to those in a previous study^3^ showing that increased mortality before the pandemic may have been the factor associated with the greatest increase mortality during the pandemic. In addition, the positive correlation between per-capita GDP and life expectancy is well known,^4^ and a negative correlation between per-capita GDP and EM was observed during the Spanish influenza pandemic.^5^ Thus, the results of this study were not unexpected.

The primary limitation of this study was its purely exploratory and descriptive nature using multiple existing data sources due to its ecological design. However, the results suggest that long life expectancy at old age in aging countries may be considered a proxy variable associated with high-quality health care systems and resilience to health care crises, including pandemics.

## Notes

Supplement. **eTable.** Definitions and Descriptions of Covariates
