# Supplementary material for: Association Between Life Expectancy at Age 60 Years Before the COVID-19 Pandemic and Excess Mortality During the Pandemic in Aging Countries
Source: JAMA Netw Open. 2022 Oct 19;5(10):e2237528. doi: 10.1001/jamanetworkopen.2022.37528 (PMC9582896; doi:10.1001/jamanetworkopen.2022.37528)
Supplement: Supplement. — eTable. Definitions and Descriptions of Covariates [file jamanetwopen-e2237528-s001.pdf]

Supplemental Online Content

Urashima M, Tanaka E, Ishihara H, Akutsu T. Association between life expectancy at age 60 years before the COVID-19 pandemic and excess mortality during the pandemic in aging countries. *JAMA Netw Open*. 2022;5(10):e2237528.
doi:10.1001/jamanetworkopen.2022.37528

eTable. Definitions and Descriptions of Covariates

This supplemental material has been provided by the authors to give readers additional information about their work.

© 2022 Urashima M et al. *JAMA Network Open.*

eTable 1. Definitions and Descriptions of Covariates

Covariates	Definition / Description / Information source	Years	URL	Access day
**Ratio of aging population**				
Population ≥ 60 years of age (%)	United Nations Population Division, New York, World Population Prospects: The 2019 Revision; supplemented by data from the United Nations Statistics Division, New York, Demographic Yearbook 2015 and Secretariat for the Pacific	2019	https://data.un.org/_Docs/SYB/CSV/SYB62_1_201907_Population,%20Surface%20Area%20and%20Density.csv	2020/05/22
**Vaccine**				
Fully vaccinated population (%)	People fully vaccinated (%) in each country at the end of 2021.	2021/12/30	https://ourworldindata.org/explorers/coronavirus-data-explorer?zoomToSelection=true&time=2020-03-01..latest&facet=none&pickerSort=asc&pickerMetric=location&Metric=Confirmed+cases&Interval=7-day+rolling+average&Relative+to+Population=true&Color+by+test+positivity=false&country=USA~GBR~CAN~DEU~ITA~IND	2022/6/14

© 2022 Urashima M et al. *JAMA Network Open.*

Covariates	Definition / Description / Information source	Years	URL	Access day
**Life expectancy**				
Life expectancy at birth (years)	Average number of years that a newborn could expect to live, if he or she were to pass through life exposed to the sex- and age-specific death rates prevailing at the time of his or her birth, for a specific year, in a given country, territory or geographic area.	2016	https://www.who.int/data/gho/data/indicators/indicator-details/GHO/life-expectancy-at-birth-(years)	2020/05/22
Life expectancy at age 60 (years)	Average number of years that a person aged 60 years old could expect to live, if he or she were to pass through life exposed to the sex- and age-specific death rates prevailing at the time of his or her 60 years, for a specific year, in a given country, territory, or geographic area.	2016	https://www.who.int/data/gho/data/indicators/indicator-details/GHO/life-expectancy-at-age-60-(years)	2020/05/22
Healthy life expectancy at birth (years)	Average number of years that a person can expect to live in "full health" by taking into account years lived in less than full health due to disease and/or injury.	2016	https://www.who.int/data/gho/data/indicators/indicator-details/GHO/healthy-life-expectancy-(hale)-at-birth-(years)	2020/05/22
Healthy life expectancy at age 60 (years)	Average number of years in full health a person (usually at age 60) can expect to live based on current rates of ill-health and mortality. Disaggregated by gender.	2016	https://www.who.int/data/gho/data/indicators/indicator-details/GHO/healthy-life-expectancy-(hale)-at-age-60-(years)	2020/05/22

© 2022 Urashima M et al. *JAMA Network Open.*

Covariates	Definition / Description / Information source	Years	URL	Access day
**Life-style**				
Prevalence of obesity among adults, BMI ≥ 25 (%)	Percentage of defined population with a body mass index (BMI) of 25 kg/m^2 or higher.	2016	https://www.who.int/data/gho/data/indicators/indicator-details/GHO/prevalence-of-overweight-among-adults-bmi-greaterequal-25-(crude-estimate)-(-)	2020/05/22
Prevalence of obesity among adults, BMI ≥ 30 (%)	Percentage of defined population with a body mass index (BMI) of 30 kg/m^2 or higher.	2016	https://www.who.int/data/gho/data/indicators/indicator-details/GHO/prevalence-of-obesity-among-adults-bmi-=-30-(crude-estimate)-(-)	2020/05/22
Alcohol drinking, total per capita consumption (APC) (in liters of pure alcohol)	Total APC is defined as the total (sum of three-year average recorded and unrecorded APC, adjusted for tourist consumption) amount of alcohol consumed per adult (15+ years) over a calendar year, in liters of pure alcohol. Recorded alcohol consumption refers to official statistics (production, import, export, and sales or taxation data), while the unrecorded alcohol consumption refers to alcohol that is not taxed and is outside the usual system of governmental control. Tourist consumption takes into account tourists visiting the country and inhabitants	2016	https://www.who.int/data/gho/data/indicators/indicator-details/GHO/alcohol-total-per-capita-(15-years)-consumption-(in-litres-of-pure-alcohol)	2020/05/22

© 2022 Urashima M et al. *JAMA Network Open.*

	visiting other countries. Positive figures denote alcohol consumption of outbound tourists being greater than alcohol consumption by inbound tourists, and negative numbers the opposite. Tourist consumption is based on UN tourist statistics.			
Prevalence of smoking any tobacco product among males aged ≥15 years (%)	Percentage of the population aged 15 years and above who currently use any tobacco product (smoked and/or smokeless tobacco) on a daily or non-daily basis. Note that most countries collect data about smoking, but not smokeless tobacco use, leaving gaps in tobacco use data, which prevent global and regional summaries of tobacco use rates. Until data improve, the estimates will reflect the percentage of the population aged 15 years and over who currently smoke.	2015	https://www.who.int/data/gho/data/indicators/indicator-details/GHO/prevalence-of-smoking-any-tobacco-product-among-persons-aged-=-15-years	2020/05/22
Prevalence of smoking any tobacco product among females aged ≥ 15 years (%)	Percentage of the population aged 15 years and above who currently use any tobacco product (smoked and/or smokeless tobacco) on a daily or non-daily basis. Note that most countries collect data about smoking, but not smokeless tobacco use, leaving gaps in tobacco use data, which prevent global and regional summaries of tobacco use rates. Until data improve, the estimates will reflect the percentage of the population aged 15 years and over who currently smoke.	2015	https://www.who.int/data/gho/data/indicators/indicator-details/GHO/prevalence-of-smoking-any-tobacco-product-among-persons-aged-=-15-years	2020/05/22

© 2022 Urashima M et al. *JAMA Network Open.*

Prevalence of insufficient physical activity among adults aged ≥ 18 years (%)	Percent of defined population attaining less than 150 minutes of moderate-intensity physical activity per week, or less than 75 minutes of vigorous-intensity physical activity per week, or the equivalent.	2016	https://www.who.int/data/gho/data/indicators/indicator-details/GHO/prevalence-of-insufficient-physical-activity-among-adults-aged-18-years-(crude-estimate)-(-)	2020/05/22
**Life-style disease prevalence**				
Prevalence of high blood pressure (systolic ≥140 or diastolic ≥90 mmHg) (%)	Percent of the defined population with high blood pressure (systolic blood pressure ≥ 140 OR diastolic blood pressure ≥ 90).	2015	https://www.who.int/data/gho/data/indicators/indicator-details/GHO/raised-blood-pressure-(sbp-=140-or-dbp-=90)-(crude-estimate)	2020/05/22
Prevalence of high fasting blood glucose (≥7.0 mmol/L or on medication) (%)	Percent of the defined population with fasting glucose ≥126 mg/dL (7.0 mmol/L) or history of diagnosis of diabetes or use of insulin or oral hypoglycemic drugs.	2014	https://www.who.int/data/gho/data/indicators/indicator-details/GHO/raised-fasting-blood-glucose-(-=-7-0-mmol-l-or-on-medication)-(crude-estimate)	2020/05/22
Prevalence of increased total cholesterol (≥5.0 mmol/L) (%)	Percentage of defined population with total cholesterol ≥ 190 mg/dL (5.0 mmol/L).	2008	https://www.who.int/data/gho/data/indicators/indicator-details/GHO/raised-total-cholesterol-(-=-5-0-mmol-l)-(crude-estimate)	2020/05/22

© 2022 Urashima M et al. *JAMA Network Open.*

Covariates	Definition / Description / Information source	Years	URL	Access day
**Death due to life-style diseases**				
Death due to chronic obstructive pulmonary disease (%)	This parameter was calculated by the number of death due to chronic obstructive pulmonary disease in adults (estimated above 25 years) per 100,000 population.	2016	https://www.who.int/data/gho/data/indicators/indicator-details/GHO/ambient-and-household-air-pollution-attributable-death-rate-(per-100-000-population)	2020/05/22
Death due to ischemic heart disease (%)	This parameter was calculated by the number of death due to ischemic heart diseases in adults (estimated above 25 years) per 100,000 population. in adults (estimated above 25 years)	2016	https://www.who.int/data/gho/data/indicators/indicator-details/GHO/ambient-and-household-air-pollution-attributable-death-rate-(per-100-000-population)	2020/05/22
Death due to lower respiratory infections (%)	This parameter was calculated by the number of death due to acute respiratory infections (estimated for all ages) per 100,000 population.	2016	https://www.who.int/data/gho/data/indicators/indicator-details/GHO/ambient-and-household-air-pollution-attributable-death-rate-(per-100-000-population)	2020/05/22

© 2022 Urashima M et al. *JAMA Network Open.*

Covariates	Definition / Description / Information source	Years	URL	Access day
Death due to stroke (%)	This parameter was calculated by the number of death due to cerebrovascular diseases in adults (estimated above 25 years) per 100,000 population.	2016	https://www.who.int/data/gho/data/indicators/indicator-details/GHO/ambient-and-household-air-pollution-attributable-death-rate-(per-100-000-population)	2020/05/22
Death due to tracheal, bronchial and lung cancers (%)	This parameter was calculated by the number of death due to lung cancer in adults (estimated above 25 years) per 100,000 population.	2016	https://www.who.int/data/gho/data/indicators/indicator-details/GHO/ambient-and-household-air-pollution-attributable-death-rate-(per-100-000-population)	2020/05/22
Ambient and household air pollution attributable death rate (n per 100,000 population)	This parameter was calculated by the number of total death due to chronic obstructive pulmonary disease, ischemic heart diseases, cerebrovascular diseases, and lung cancer in adults (estimated above 25 years) as well as acute respiratory infections (estimated for all ages) per 100,000 population	2016	https://www.who.int/data/gho/data/indicators/indicator-details/GHO/ambient-and-household-air-pollution-attributable-death-rate-(per-100-000-population)	2020/05/22
Annual mean concentration of particulate matter less than 2.5 microns in diameter (PM2.5)	Mean annual concentration of fine suspended particles of less than 2.5 microns diameter is a common measure of air pollution. The mean is a population-weighted average for urban population in a country.	2016	https://www.who.int/data/gho/data/indicators/indicator-details/GHO/concentrations-of-fine-particulate-matter-(pm2-5)	2020/05/22

© 2022 Urashima M et al. *JAMA Network Open.*

Covariates	Definition / Description / Information source	Years	URL	Access day
**Mortality by age group**				
Neonatal mortality rate (n per 1000 live births)	Number of deaths during the first 28 completed days of life per 1000 live births in a given year or other periods. Neonatal deaths (deaths among live births during the first 28 completed days of life) can be subdivided into early neonatal deaths, occurring during the first 7 days of life, and late neonatal deaths, occurring after the 7th day but before the 28th completed day of life.	2018	https://www.who.int/data/gho/data/indicators/indicator-details/GHO/neonatal-mortality-rate-(per-1000-live-births)	2020/05/22
Infantile mortality rate (n per 1000 live births)	Probability of a child born in a specific year or period dying before reaching the age of five, if subject to age-specific mortality rates of that period. Under-five mortality rate as defined here is strictly speaking not a rate (i.e. the number of deaths divided by the number of population at risk during a certain period of time), but the probability of death derived from a life table and expressed as rate per 1000 live births.	2018	https://www.who.int/data/gho/data/indicators/indicator-details/GHO/under-five-mortality-rate-(probability-of-dying-by-age-5-per-1000-live-births)	2020/05/22

© 2022 Urashima M et al. *JAMA Network Open.*

Covariates	Definition / Description / Information source	Years	URL	Access day
Under-five mortality rate (probability of dying by age 5 per 1000 live births)	Probability of a child born in a specific year or period dying before reaching the age of five, if subject to age-specific mortality rates of that period. Under-five mortality rate as defined here is strictly speaking not a rate (i.e. the number of deaths divided by the number of population at risk during a certain period of time), but a probability of death derived from a life table and expressed as rate per 1000 live births.	2018	https://www.who.int/data/gho/data/indicators/indicator-details/GHO/under-five-mortality-rate-(probability-of-dying-by-age-5-per-1000-live-births)	2020/05/22
Mortality rate for 5-14 years of age (probability of dying per 1000 children aged 5-14 years)	Probability that a child aged 5 dies before reaching his/her 15th birthday.	2018	https://www.who.int/data/gho/data/indicators/indicator-details/GHO/mortality-rate-for-5-14-year-olds-(probability-of-dying-per-1000-children-aged-5-14-years)	2020/05/22
Adult mortality rate (probability of dying between 15 and 60 years of age per 1000 population)	Probability that a 15-year-old person will die before reaching his/her 60th birthday. The probability of dying between the ages of 15 and 60 years (per 1 000 population) per year among a hypothetical cohort of 100,000 people that would experience the age-specific mortality rate of the reporting year.	2016	https://www.who.int/data/gho/data/indicators/indicator-details/GHO/adult-mortality-rate-(probability-of-dying-between-15-and-60-years-per-1000-population)	2020/05/22

© 2022 Urashima M et al. *JAMA Network Open.*

Covariates	Definition / Description / Information source	Years	URL	Access day
Probability of dying between age 30 and exact age 70 from any of cardiovascular disease, cancer, diabetes, or chronic respiratory diseases	Per cent of 30-year-old-people who would die before their 70th birthday from any of cardiovascular disease, cancer, diabetes, or chronic respiratory diseases, assuming that she/he would experience current mortality rates at every age and she/he would not die from any other cause of death (e.g., injuries or HIV/AIDS).	2016	https://www.who.int/data/gho/data/indicators/indicator-details/GHO/probability-(-)-of-dying-between-age-30-and-exact-age-70-from-any-of-cardiovascular-disease-cancer-diabetes-or-chronic-respiratory-disease	2020/05/22
**Population and dynamics**				
Population (in million)	Elaboration of data by United Nations, Department of Economic and Social Affairs, Population Division. World Population Prospects: The 2019 Revision (Medium-fertility variant).	2020	https://www.worldometers.info/world-population/population-by-country/	2020/05/22

© 2022 Urashima M et al. *JAMA Network Open.*

Covariates	Definition / Description / Information source	Years	URL	Access day
Yearly change in population (x 10^{-4}%)	Elaboration of data by United Nations, Department of Economic and Social Affairs, Population Division. World Population Prospects: The 2019 Revision (Medium-fertility variant).	2020	https://www.worldometers.info/world-population/population-by-country/	2020/05/22
Net change in population (n x 10^{-3})				
Density (km^2)				
Land area (km^2 x 10^3)				
Migrants, net (n x 10^3)				
Fertility rate (n)				
Median age (years old)				
Urban population percentage (%)				

© 2022 Urashima M et al. *JAMA Network Open.*

Covariates	Definition / Description / Information source	Years	URL	Access day
**Economics**				
Gross domestic product (GDP) (billions of USD)	United Nations Statistics Division, New York, National Accounts Statistics: Analysis of Main Aggregates (AMA) database, last accessed February 2018.	2017	https://data.un.org/_Docs/SYB/CSV/SYB62_230_201904_GDP%20and%20GDP%20Per%20Capita.csv	2020/05/22
GDP per capita (USD 1000)	United Nations Statistics Division, New York, National Accounts Statistics: Analysis of Main Aggregates (AMA) database, last accessed February 2018.	2017	https://data.un.org/_Docs/SYB/CSV/SYB62_230_201904_GDP%20and%20GDP%20Per%20Capita.csv	2020/05/22
Total unemployment (%)	International Labour Organization (ILO), Geneva, Key Indicators of the Labour Market (KILM 9th edition) and the ILOSTAT database, last accessed January 2019.	2019	https://data.un.org/_Docs/SYB/CSV/SYB62_329_201904_Labour%20Force%20and%20Unemployment.csv	2020/05/22
Male unemployment (%)	International Labour Organization (ILO), Geneva, Key Indicators of the Labour Market (KILM 9th edition) and the ILOSTAT database, last accessed January 2019.	2019	https://data.un.org/_Docs/SYB/CSV/SYB62_329_201904_Labour%20Force%20and%20Unemployment.csv	2020/05/22
Female unemployment (%)				
Labor force total participation (%)				
Labor force male participation (%)				
Labor force female participation (%)				

© 2022 Urashima M et al. *JAMA Network Open.*

Covariates	Definition / Description / Information source	Years	URL	Access day
**National Health policy**				
International health regulation (IHR) score (%)	Percentage of attributes of 13 core capacities that have been attained at a specific point in time. The 13 core capacities are: (1) National legislation, policy and financing; (2) Coordination and National Focal Point communications; (3) Surveillance; (4) Response; (5) Preparedness; (6) Risk communication; (7) Human resources; (8) Laboratory; (9) Points of entry; (10) Zoonotic events; (11) Food safety; (12) Chemical events; (13) Radionuclear emergencies.	2014-2019	https://www.who.int/data/gho/data/indicators/indicator-details/GHO/-average-of-13-international-health-regulations-core-capacity-scores-1st-version-of-the-questionnaire	2020/05/22
Universal health coverage (UHC) index	Percentage of tracer indicators in the UHC service coverage index with primary country data sources between 2013-2017.	2013-2017	https://www.who.int/data/gho/data/indicators/indicator-details/GHO/data-availability-for-uhc-index-of-essential-service-coverage-(-)	2020/05/22
Hospital beds (n) per 10,000 population	Number of hospital beds available per 10,000 inhabitants in a population.	2004-2015	https://www.who.int/data/gho/data/indicators/indicator-details/GHO/hospital-beds-(per-10-000-population)	2020/05/22

© 2022 Urashima M et al. *JAMA Network Open.*

Covariates	Definition / Description / Information source	Years	URL	Access day
Medical doctors (n) per 10,000 population	Includes generalists, specialist medical practitioners and medical doctors not further defined, in the given national and/or subnational area. Depending on the nature of the original data source, it may include practicing (active) physicians only or all registered physicians. The ISCO -08 codes included here are 221, 2211 and 2212 </Definition> <Associated Terms name="Associated terms">. The WHO framework for classifying health workers draws on the latest revisions of international classifications, including the International Standard Classification of Occupations (revision 2008), the International Standard Classification of Education (revision 2011) and the International Standard Industrial Classification of All Economic Activities (revision 4).	2001-2018	https://www.who.int/data/gho/data/indicators/indicator-details/GHO/medical-doctors-(per-10-000-population)	2020/05/22

© 2022 Urashima M et al. *JAMA Network Open.*

Covariates	Definition / Description / Information source	Years	URL	Access day
Nursing midwifery (n) per 10,000 population	Number of nursing and midwifery personnel includes nursing personnel and midwifery personnel in the given national and/or subnational area. Depending on the nature of the original data source, it may include practicing (active) nursing and midwifery personnel only or all registered nursing and midwifery personnel The ISCO -08 codes included here are 2221, 2222, 3221 and 3222.	2013-2018	https://www.who.int/data/gho/data/indicators/indicator-details/GHO/nursing-and-midwifery-personnel-(per-10-000-population)	2020/05/22
Total expenditure on health as a percentage of GDP (%)	Level of total expenditure on health (THE) expressed as a percentage of gross domestic product (GDP).	2014	https://www.who.int/data/gho/data/indicators/indicator-details/GHO/total-expenditure-on-health-as-a-percentage-of-gross-domestic-product	2020/05/22
Population with household expenditures on health greater than 10% of total household expenditure or income (%)	Proportion of the population with household expenditure on health exceeding 10% of total household expenditure or income.	1993-2018	https://www.who.int/data/gho/data/indicators/indicator-details/GHO/population-with-household-expenditures-on-health-greater-than-10-of-total-household-expenditure-or-income-(sdg-3-8-2)-(-)	2020/05/22

© 2022 Urashima M et al. *JAMA Network Open.*
